# Loss of perceived social role, an index of social frailty, is an independent predictor of future adverse events in hospitalized patients with heart failure

**DOI:** 10.3389/fcvm.2022.1051570

**Published:** 2022-12-20

**Authors:** Ayako Watanabe, Satoshi Katano, Toshiyuki Yano, Ryohei Nagaoka, Ryo Numazawa, Suguru Honma, Kotaro Yamano, Yusuke Fujisawa, Katsuhiko Ohori, Hidemichi Kouzu, Tomoyuki Ishigo, Masaki Katayose, Akiyoshi Hashimoto, Masato Furuhashi

**Affiliations:** ^1^Division of Nursing, Sapporo Medical University Hospital, Sapporo, Japan; ^2^Division of Rehabilitation, Sapporo Medical University Hospital, Sapporo, Japan; ^3^Department of Cardiovascular, Sapporo Medical University School of Medicine, Renal and Metabolic Medicine, Sapporo, Japan; ^4^Graduate School of Medicine, Sapporo Medical University, Sapporo, Japan; ^5^Department of Rehabilitation, Sapporo Cardiovascular Hospital, Sapporo, Japan; ^6^Second Division of Physical Therapy, Sapporo Medical University School of Health Science, Sapporo, Japan; ^7^Department of Cardiology, Hokkaidō Cardiovascular Hospital, Sapporo, Japan; ^8^Division of Hospital Pharmacy, Sapporo Medical University Hospital, Sapporo, Japan; ^9^Division of Health Care Administration and Management, Sapporo Medical University School of Medicine, Sapporo, Japan

**Keywords:** social frailty, social role, feeling of usefulness, heart failure, prognosis, older

## Abstract

**Aims:**

Although the impact of physical frailty on prognosis and the effect of cardiac rehabilitation in HF patients has been well established, data for the prognostic impact of social frailty (SF) in HF patients are limited. In addition, the relative importance of each SF domain in clinical outcomes remains unclear. We aimed to get a new insight into the associations of SF with clinical outcomes in elderly hospitalized HF patients.

**Methods:**

A single-center, retrospective cohort study was conducted using data from 310 in-hospital HF patients aged ≥ 65 years (mean age of 78 ± 8 years; 49% women). Makizako’s five questions, a self-reported questionnaire, were used to define SF. The primary outcome was composite events defined by all-cause death and cardiovascular events.

**Results:**

Of the 310 elderly HF patients, 188 patients (61%) had SF. Seventy-five patients (24%) had composite events during a mean follow-up period of 1.93 ± 0.91 years. Kaplan-Meier curves showed that patients with SF had a significantly higher composite event rate than patients without SF. In multivariate Cox regression analyses, SF was independently associated with a higher composite event rate after adjusting for prognostic markers [adjusted hazard ratio (HR), 2.01; 95% confidence interval (CI), 1.07–3.78; *p* = 0.04]. Of the 5 questions for defining SF, an answer of yes to the question about not feeling helpful toward friends or family, which indicates loss of perceived social role, was an independent predictor of composite events (adjusted HR, 2.28; 95% CI, 1.36–3.82; *p* < 0.01). Inclusion of loss of perceived social role into the baseline prognostic model improved both the continuous net reclassification improvement (0.562; 95% CI, 0.298–0.827; *p* < 0.01) and integrated discrimination improvement (0.031; 95% CI, 0.006–0.056; *p* = 0.02).

**Conclusion:**

Loss of perceived social role is associated with increased adverse event risk and provides additive prognostic information in elderly HF patients.

## Introduction

Heart failure (HF) is a serious public health problem affecting about 26 million people worldwide and with substantial medical costs (1, 2). The prevalence of HF increases with advance of age: more than 80% of patients diagnosed with HF are over 65 years of age, and the prevalence of HF in older people has been rapidly increasing in most countries throughout the world (1). Despite significant advances in medical therapies and prevention strategies, readmission rates remain high, particularly for older HF patients (3). As a result, their overall functionality deteriorates over time (4), leading to a high level of functional dependence, poor quality of life, and high rate of mortality and morbidity (5). Importantly, cardiac dysfunction is not a sole mechanism of functional dependence and poor quality of life in older HF patient: Extra-cardiac factors such as chronic kidney disease (CKD), anemia, malnutrition, and cachexia are involved in the mechanism (6, 7). Thus, a better understanding of the multidimensional features is required to establish appropriate preventive and management programs for older HF patients.

Frailty is a clinical syndrome characterized by an age-dependent increase in vulnerability to physiological/psychological stresses, leading to an increased likelihood of adverse clinical outcomes such as disability, hospitalization, and mortality (8, 9). Frailty has been recognized as a multidimensional concept that includes physical, psychological, and social domains, which are intertwined with each other. Although it is difficult to unveil the complex relationships among these domains, the results of an observational study in community-dwelling older people showed that the presence of social frailty (SF), a concept encompassing loss of social roles, social networks, and social activity, may be an antecedent event of physical and psychological frailty (10). Physical frailty frequently coexists with HF, the presence of which has been shown to be associated with worse prognosis, increased rehospitalization rate, and poor response to comprehensive cardiac rehabilitation (11, 12), but available data on the prevalence of SF and its prognostic impact in HF patients have been limited. To our knowledge, there has been only one study showing the prognostic impact of SF in hospitalized older HF patients: the presence of SF was shown to be independently associated with increased occurrence of all-cause death and HF hospitalization after discharge in a multicenter prospective cohort study (13). However, the mechanisms of the close associations between SF and clinical outcomes remain unclear. This is a critical issue for establishing an SF-targeted management strategy in older HF patients.

In the present study, we used Makizako’s five questions, a well-established questionnaire for defining SF, and examined the prognostic impact of SF and which domains of SF are associated with poor clinical outcome in older hospitalized HF patients.

## Materials and methods

### Study design and study subjects

This study was a single-center, retrospective cohort study. We reviewed data for a cohort of consecutive HF patients admitted to our institute for HF treatment between March 1, 2015 and December 31, 2020 since routine assessment of SF was commenced from March 1, 2015. HF was diagnosed according to the Japanese Circulation Society/Japanese HF Society Guidelines for HF. The inclusion criteria were (1) patients who were aged ≥ 65 years and (2) patients who underwent cardiac rehabilitation programs and multidisciplinary intervention, including education on self-monitoring and medications as well as nutritional guidance by a HF team consisting of cardiologists, nurses, physical therapists, pharmacists, dietitians, and social workers. The cardiac rehabilitation program was performed as described previously (6). Patients who were lost to follow-up within 6 months after discharge were excluded from the analyses.

This study was conducted in strict adherence to the principles of the Declaration of Helsinki and was approved by the Clinical Investigation Ethics Committee of Sapporo Medical University Hospital (Number 302–243).

### Assessment of social frailty

Trained personnel assessed SF using Makizako’s five questions, which have been validated by Makizako et al. (14) This questionnaire was originally derived from community-dwelling older adults and is associated with muscle weakness (15) and new onset of physical frailty (16) and disability (14) involving the following five items: (1) “Do you go out less frequently compared with last year?” (2) “Do you sometimes visit your friends?” (3) “Do you feel you are helpful to friends or family?” (4) “Do you live alone?” and (5) “Do you talk with someone every day?”. Answers of “yes” to the questions 1 and 4 and “no” to the questions 2, 3, and 5 were considered negative responses (Supplementary Table 1). SF was defined as two or more negative responses to the above questions. (14)

### Collection of data for other clinical parameters

All data for variables, including demographic data, medications, laboratory data, echocardiographic data, physical performance and functional status, nutritional status, comorbidities, and physical frailty, were collected from the patients’ medical records.

Laboratory data for N-terminal pro-brain natriuretic peptide (NT-proBNP), serum albumin, hemoglobin, cystatin C, and cystatin C-based glomerular filtration rate (eGFRcys) were obtained within 7 days before discharge. eGFRcys was calculated using an equation developed for Japanese individuals (17). CKD was defined as eGFRcys < 60 mL/min/1.73 m^2^. Transthoracic echocardiography was performed by the standard protocol, and left ventricular ejection fraction (LVEF) was measured by the modified Simpson method. Heart failure with reduced ejection fraction (HFrEF) and heart failure with preserved ejection fraction (HFpEF) were defined as LVEF of < 40 and ≥ 50%, respectively.

The 10-m gait speed and handgrip strength were used to measure physical performance. In measuring the usual 10-m gait speed, the patients were asked to walk at their usual speed over the middle 10 m of a 16-m walkway as previously described (18). Patients were allowed to use canes or other walking aids during the test. Handgrip strength was measured with a Smedley spring-type digital dynamometer (TKK 5401 GRIP-D, TAKEI, Niigata, Japan): patients stood with their upper limbs along the sides of their trunk and performed the test alternately with their right and left hands. The absolute value of the maximum reading of the two trials using both hands was calculated (kg). As previously described (6, 19), functional status for performing basic activities of daily living (ADL) was assessed using the Barthel Index by trained physical therapists over a period of 3 days before discharge. Complete dependence and complete independence are indicated by Barthel Index scores of 0 and 100, respectively.

As described previously, nutritional status was assessed before discharge by a mini nutritional assessment short form (MNA-SF) (6, 20, 21). The MNA-SF consists of 6 questions about reducing food intake over the past 3 months, weight loss during the past 3 months, mobility, psychological stress or acute disease in the past 3 months, neuropsychological problems, and BMI, and it is scored 0–14.

The existence of comorbidities was assessed on the basis of medical information, including the patient’s history, data for parameters in clinical examinations, and prescribed drugs. As described previously, comorbidities were assessed using the Charlson comorbidity index (CCI) (20, 22). Cognitive function was evaluated using the Mini-Cog (23), a composite of a 3-item recall test and a clock-drawing test, by trained personnel. The Mini-Cog was scored on a 5-point scale (1 point for each correct word recalled and 2 points for correct clock drawing), and a score ≤ 2 was considered as indicating cognitive impairment.

Physical frailty was measured using the revised Japanese version of the Cardiovascular Health Study (CHS) criteria (24) by a trained physical therapist. Patients with physical frailty were defined as patients who had at least three of the following characteristics: weakness (handgrip strength ≤ 28 kg in men or ≤ 18 kg in women), shrinking (unintentional loss of two or more kg in the past 6 months), exhaustion (feeling tired without reason in the past 2 weeks), slowness (gait speed < 1.0 m/s), and low activity (no engagement in moderate levels of physical exercise or sports aimed at health or no engagement in a low level of physical exercise aimed at health).

### Clinical endpoints

The clinical endpoint in this study was the first adverse event defined as a composite of all-cause death and unscheduled readmission due to worsening HF. An episode of worsening HF was defined as either an unplanned scheduled hospitalization for HF or an urgent visit due to worsening HF symptoms. Data for clinical endpoints in the patients were obtained for up to 3 years after enrollment of the first patient and 1 year after enrollment of the last patient.

### Statistical analysis

Data are presented as means ± standard deviation or medians [interquartile range (IQR): 25th—75th percentiles] depending on the results of the Shapiro-Wilk test for normality of data distribution. Categorical data are expressed as numbers with percentages. As appropriate, the baseline characteristics were compared by Welch’s *t*-test, the Mann-Whitney U test, or the chi-square test. Survival curves were calculated by the Kaplan-Meier method, and the statistical significance of differences between the curves was assessed by log-rank statistics. Univariate and multivariate Cox proportional hazards analyses were used to evaluate prognostic predictive ability. Multivariate Cox proportional hazard model was adjusted for age, sex, logarithmic NT-proBNP (log NT-proBNP), eGFRcys, HFrEF, prior hospital admission due to HF, and CCI. Then, NYHA functional class 3, physical frailty, physical function (10-m gait speed and hand grip strength), cognitive function (Mini-cog), or nutritional status indicator (BMI, MNA-SF score ≤ 7, serum albumin concentration, and hemoglobin levels) were further added into the multivariate model, respectively. To examine whether information on SF or each item in the questionnaire provides a significant incremental prognostic value over that for the pre-existing risk factors, we constructed several models: a baseline model including the variables used for the adjustment in the abovementioned Cox proportional hazard models and expanded models including the variables in the baseline model plus the presence/absence of SF or negative/positive response to each item in the questionnaire. The continuous net reclassification improvement (cNRI) and integrated discrimination improvement (IDI)—sensitive statistical methods to quantify improvement of a model when a new variable is added—were used to calculate the increase in information by SF and response to each item in the questionnaire compared to a baseline model (25, 26). Missing data were imputed using a multiple imputation analysis. The imputation model included the outcome and all exposures and adjustment variables. Assuming missing at random, multiple imputations were performed using chained equations with *M* = 100 imputations to construct the multivariate Cox proportional hazard model in each imputed dataset and the estimates were pooled to obtain HR s adjusted for covariates following Rubin’s rule (27). Similarly, we computed cNRI and IDI in each imputed dataset and pooled them. For sensitivity analysis, univariate and multivariate Cox proportional hazard analyses were performed for patients with a complete dataset. A two-tailed *p*-value < 0.05 was considered statistically significant. Statistical analyses were performed using JMP Pro version 15.2.1 (SAS Institute Inc., Cary, NC, USA) and R version 4.1.2 R Foundation for Statistical Computing, Vienna.^1^

## Results

**FIGURE 1:**
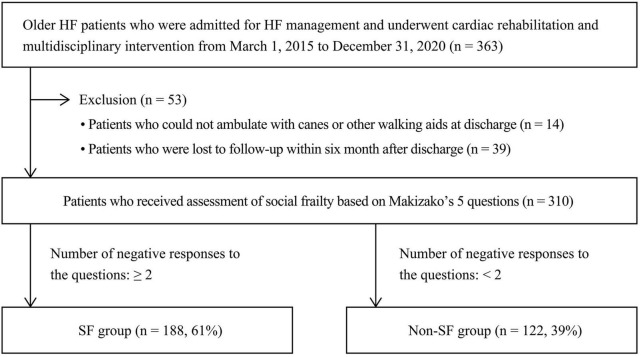
Flow chart of the inclusion of study subjects. HF, heart failure; SF, social frailty.

Of 362 patients initially screened, 53 were excluded by the exclusion criteria. Thus, data for 310 patients were used for analyses (Figure 1).

### Baseline characteristics and prevalence of social frailty

As shown in Table 1, the median age of the patients was 79 years (IQR, 72, 84 years), and 46% of the patients were women. Thirty-six percent of the patients were classified as NYHA functional class III. The median LVEF was 51.1% (IQR, 35.1, 63.5%), and 30 and 54% of the patients had HFrEF and HFpEF, respectively. Forty-two percent of the patients had a history of HF hospitalization. The most frequent etiology of HF was valvular heart disease (42%), followed by cardiomyopathy (25%) and ischemic heart disease (16%).

**TABLE 1:** Baseline characteristics.

	Missing	Overall	SF group	Non-SF group	*P*-value
	*n* (%)	*n* = 310	*n* = 188	*n* = 122	
Age, *years*		79(72,84)	80(73,86)	76(71,82)	<0.01
Women, *n (%)*		143 (46)	95 (51)	72 (59)	0.14
Height, *cm*		157 ± 10	157 ± 10	159 ± 9	0.03
Body weight, *kg*		55.4(47.0,61.6)	52.2(44.3,60.5)	57.7(50.8,63.1)	<0.01
BMI, *kg/m^2^*		21.8(19.5,23.9)	21.6(19.1,23.6)	22.3(20.4,24.1)	0.02
Systolic blood pressure, *mmHg*		117(105,131)	117(106,130)	118(104,132)	0.99
**NYHA functional class, *n (%)***					
I		16 (5)	8 (4)	8 (7)	0.09
II		183 (59)	104 (55)	79 (65)	
III		111 (36)	76 (40)	35 (29)	
LVEF,%		51.1(35.1,63.5)	51.8(35.2,64.4)	50.1(34.8,62.8)	0.44
HFrEF, *n (%)*		94 (30)	57 (30)	37 (31)	0.36
HFpEF, *n (%)*		167 (54)	106 (56)	61 (50)	
Prior hospital admission due to HF, *n (%)*		129 (42)	86 (46)	43 (35)	0.07
**Etiology, *n (%)***					
Cardiomyopathy		77 (25)	39 (21)	38 (31)	0.19
Valvular heart disease		130 (42)	82 (44)	48 (39)	
Ischemic		50 (16)	31 (16)	19 (16)	
**Comorbidity, *n (%)***					
Hypertension		217 (70)	134 (71)	83 (68)	0.54
Dyslipidemia		188 (61)	106 (56)	82 (67)	0.06
Diabetes mellitus		127 (41)	76 (40)	51 (42)	0.81
Atrial fibrillation		126 (41)	72 (38)	54 (44)	0.30
Cancer		84 (27)	51 (27)	33 (27)	0.99
Charlson comorbidity index, *points*		3(2,4)	3(2,4)	3(2,3)	0.07
**Physical function**					
10-m gait speed, *m/sec*	6 (2)	0.831 ± 0.276	0.788 ± 0.280	0.898 ± 0.258	<0.01
Hand grip strength, *kg*	23 (7)	21.1(15.1,27.9)	19.5(13.9,26.7)	23.6(17.6,30.0)	<0.01
Barthel Index, *points*		90(80,95)	85(75,95)	90(85,95)	<0.01
Physical frailty, *n (%)*	27 (9)	156 (55)	106 (66)	50 (45)	<0.01
**Nutritional Status**					
MNA-SF score		9(7,11)	8(6,10)	9(7,11)	<0.01
**Laboratory data**					
NT-proBNP, *pg/mL*		1,192(507,2,749)	1,246(498,2,867)	1,069(527,2,078)	0.29
Albumin, *g/dL*		3.5(3.2,3.7)	3.5(3.2,3.7)	3.6(3.4,3.8)	<0.01
Hemoglobin, *g/dL*		11.4(10.4,12.9)	11.1(10.2,12.3)	12.0(10.9,13.6)	<0.01
Cystatin C	20 (6)	1.24(1.04,1.68)	1.31(1.05,1.89)	1.15(1.01,1.54)	0.03
eGFRcys, *mL/min/1.73m^2^*	20 (6)	50.3(34.9,64.8)	46.2(31.8,62.3)	56.0(39.2,66.4)	<0.01
**Medication, *n (%)***					
β blocker		193 (62)	112 (60)	81 (66)	0.23
ACE-I or ARB		170 (55)	100 (53)	70 (57)	0.47
MRA		139 (45)	77 (41)	62 (51)	0.09
Loop diuretics		195 (63)	117 (62)	78 (64)	0.76
**Socioenvironmental status**					
Makizako’s social frailty score, *points*		2(1,3)	2(2,3)	1(1,1)	<0.01
Doyou go out less frequently compared with last year? (yes), *n (%)*		211 (68)	161 (86)	50 (41)	<0.01
Doyou sometimes visit your friends? (no), *n (%)*		202 (65)	166 (88)	36 (30)	<0.01
Doyou feel you are helpful to friends or family? (no), *n (%)*		81 (26)	80 (43)	1 (1)	<0.01
Doyou live alone? (yes), *n (%)*		64 (21)	53 (28)	11 (9)	<0.01
Doyou talk with someone every day? (no), *n (%)*		36 (12)	36 (19)	0 (0)	<0.01
Cohabitants, yes, *n (%)*		246 (79)	135 (72)	111 (91)	<0.01
Long-term care insurance, *n (%)*		129 (42)	103 (55)	26 (21)	<0.01
**Cognitive function**					
Mini-cog score, *point*	7 (2)	5(4,5)	5(4,5)	5(4,5)	0.06
Cognitive impairment, *n (%)*	7 (2)	31 (10)	24 (13)	7 (6)	0.04

Data are presented as mean ± standard deviation of the mean, median (interquartile range, 25th, 75th percentile), or number (with percentage). n, number of patients for whom the parameter was available. SF, social frailty; BMI, body mass index; NYHA, New York Heart Association; LVEF, left ventricular ejection fraction; HFrEF, heart failure with reduced ejection fraction; HFpEF, heart failure with preserved ejection fraction; HF, heart failure; MNA-SF, mini nutritional assessment short form; NT-proBNP, N-terminal pro B-type natriuretic peptide; eGFRcys, cystatin C-based estimated glomerular filtration rate; ACE-I, angiotensin-converting enzyme inhibitor; ARB, angiotensin receptor blocker; MRA, mineralocorticoid receptor antagonist.

Among the enrolled patients, 188 (61%) had SF. Patients with SF were significantly older than patients without SF and had lower BMI, slower gait speed, and weaker hand grip strength than did patients without SF. Barthel Index score, MNA-SF score, serum albumin concentrations, hemoglobin levels, and eGFRcys were significantly lower and serum cystatin C levels and percentage of patients with physical frailty and cognitive impairment were higher in patients with SF than in patients without SF.

### Differences between characteristics of patients with complete data and those of patients with missing data

Missing values across the seven variables varied between 2 and 9%. Fifty-nine of 310 records (19%) were incomplete. Patients with missing data had a significantly lower systolic blood pressure, LVEF, and Barthel Index score, slower gait speed, higher frequency of HFrEF, and higher frequency of history of HF hospitalization (Supplementary Table 2). Therefore, we used multiple imputations using chained equations to create and analyze 100 multiply imputed datasets, and we combined estimates and standard errors using Rubin’s rules in the next section.

### Associations of social frailty and each item of Makizako’s 5 questions with prognosis

**FIGURE 2:**
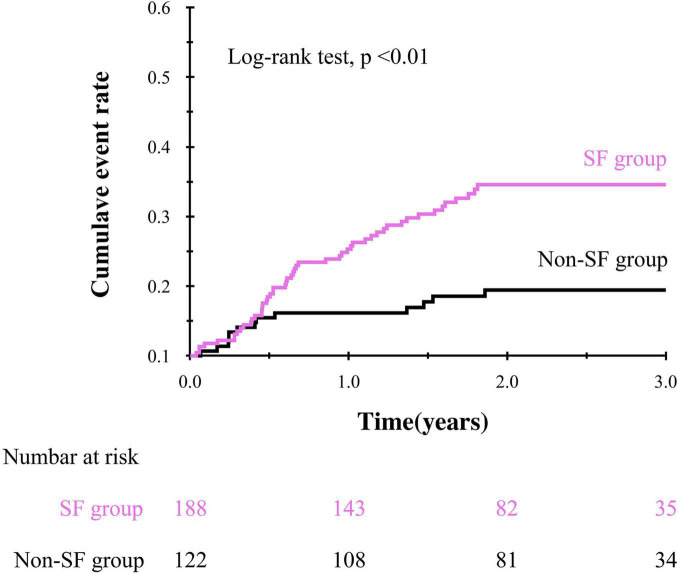
Kaplan-Meier curve showing the impact of social frailty on the composite event in older HF patients.

During the mean follow-up period of 1.93 ± 0.91 years, 64 patients (21%) experienced composite events. Kaplan-Meier survival curves showed a significantly higher cumulative composite event rate in patients with SF than in patients without SF (log-rank test, *p* < 0.01, Figure 2). Furthermore, univariate Cox proportional hazard analysis showed a significantly higher HR for the composite events in patients with SF than in patients without SF (HR, 2.84; 95% CI, 1.55–5.23; *p* < 0.01). The prognostic effect of SF remained even after adjusting for the established prognostic markers of HF in a multivariate model after multiple imputations (Table 2).

**TABLE 2:** Univariate and multivariate Cox proportional hazard analysis for composite event according to the presence/absence of social frailty.

	SF group (vs. Non-SF group) multiple imputed case			SF group (vs. Non-SF group) complete case, *n* = 259		
	HR	(95% CI)	*P*-value	HR	(95% CI)	*P*-value
Univariate model	2.84	(1.55, 5.23)	< 0.01	4.08	(1.83, 9.11)	< 0.01
Multivariate model	2.01	(1.07, 3.78)	0.04	2.86	(1.24, 6.58)	0.01
+ NYHA functional class III	2.00	(1.07, 3.77)	0.04	2.88	(1.25, 6.62)	0.01
+Physical frailty	2.02	(1.07, 3.80)	0.03	2.81	(1.22, 6.46)	0.02
+ Barthel Index score	1.94	(1.03, 3.65)	0.046	2.85	(1.24, 6.57)	0.01
+10-m gait speed	2.03	(1.08, 3.82)	0.03	2.87	(1.25, 6.59)	0.01
+ Hand grip strength	1.98	(1.05, 3.72)	0.04	2.82	(1.22, 6.49)	0.02
+Mini-cog score	2.01	(1.07, 3.77)	0.04	2.77	(1.20, 6.42)	0.02
+ BMI	1.76	(0.93, 3.35)	0.09	2.54	(1.09, 5.64)	0.03
+Malnutrition (MNA-SF score ≤ 7)	1.97	(1.05, 3.72)	0.04	2.83	(1.23, 6.52)	0.01
+ Albumin	2.02	(1.07, 3.82)	0.03	2.93	(1.27, 6.77)	0.01
+ Hemoglobin	1.94	(1.03, 3.68)	0.046	2.73	(1.18, 6.34)	0.02

The multivariate model was adjusted for age, sex, prior hospital admission due to HF, HFrEF, log NT-proBNP, eGFRcys, and Charlson comorbidity index. SF, social frailty; HF, heart failure; HR, hazard ratio; CI, confidence interval; HFrEF, heart failure with reduced ejection fraction; log NT-proBNP, logarithmic N-terminal pro B-type natriuretic peptide; eGFRcys, cystatine C-based estimated glomerular filtration rate; BMI, body mass index; MNA-SF, mini nutritional assessment short form.

**FIGURE 3:**
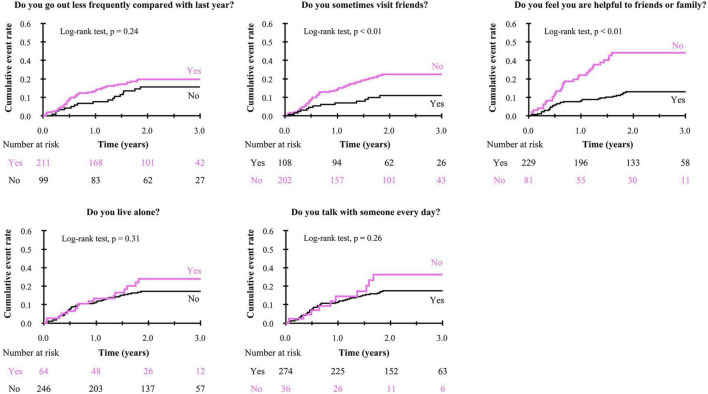
Kaplan-Meier curve for the composite event in negative/positive response to each of Makizako’s 5 questions.

**FIGURE 4:**
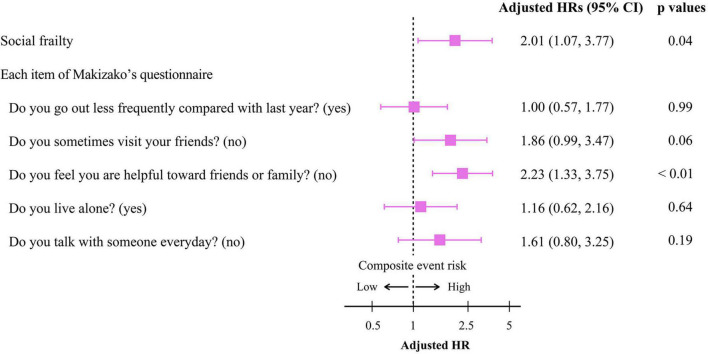
Cox proportional hazard analyses for the composite event according to the presence/absence of social frailty and the negative/positive response to each Makizako’s 5 questions in older HF patients. The model was adjusted for age, sex, prior hospital admission due to HF, HFrEF, Log NT-proBNP, eGFRcys, and Charlson comorbidity index. HR, hazard ratio; CI, confidence interval; HFrEF, heart failure with reduced ejection fraction; Log NT-proBNP, logarithmic N-terminal pro B-type natriuretic peptide; eGFRcys, cystatin C-based estimated glomerular filtration rate.

To get insights into the mechanism by which the presence of SF is associated with poor clinical outcomes, we further analyzed the impact of responses to the items in Makizako’s 5 questions on the composite events. As shown in Figure 3, higher cumulative composite event rates were found in patients who had negative responses to the questions regarding “Do you sometimes visit your friends?” and “Do you feel you are helpful toward friends or family?” than in patients who had positive responses no to those questions, respectively. Multivariate Cox proportional hazard analysis after multiple imputations showed that an answer of no to the question, “Do you feel you are helpful toward friends or family?”, which indicates loss of perceived social role, is associated with increased HR for the composite events (adjusted HR, 2.23; 95% CI, 1.33–3.75; *p* < 0.01; Figure 4). An independent association between loss of perceived social role and higher cumulative composite event rates was confirmed by sensitivity analyses in the patients with complete data (Table 2 and Supplementary Table 3).

### Incremental prognostic values of social frailty and each item of Makizako’s 5 questions

The addition of SF to the baseline model improved only cNRI (0.480; 95% CI, 0.247–0.713; *p* < 0.01), whereas inclusion of loss of perceived social role to the baseline model significantly improved both cNRI and IDI (Table 3). Among Makizako’s 5 questions, only loss of perceived social role was associated with all indexes of physical and psychological frailty, i.e., Barthel index score, gait speed, handgrip strength, and Mini-cog score, in the present study (Supplementary Table 4).

**TABLE 3:** Predictive ability for the composite event.

Models	cNRI	(95% CI)	*P*-value	IDI	(95% CI)	*P*-value
**Baseline model**						
+ Social frailty	0.480	(0.247, 0.713)	<0.01	0.017	(−0.0006, 0.035)	0.06
+ Do you go out less frequently compared with last year? (yes)	–0.049	(−0.400, 0.302)	1.00	0.000	(−0.0001, 0.0001)	0.79
+ Do you sometimes visit friends? (no)	0.366	(0.134, 0.598)	<0.01	0.016	(0.001, 0.031)	0.04
+ Do you feel you are helpful toward friends or family? (no)	0.562	(0.298, 0.827)	<0.01	0.031	(0.006, 0.056)	0.02
+ Do you live alone? (yes)	0.097	(−0.152, 0.346)	0.44	0.002	(−0.002, 0.006)	0.43
+Do you talk with someone everyday? (no)	0.219	(−0.005, 0.443)	0.06	0.005	(−0.005, 0.014)	0.33

Baseline model included age, sex, prior hospital admission due to HF, HFrEF, log NT-proBNP, eGFRcys, and Charlson comorbidity index. HF, heart failure; HFrEF, heart failure with reduced ejection fraction; log NT-proBNP, logarithmic N-terminal pro B-type natriuretic peptide; eGFRcys, cystatine C-based estimated glomerular filtration rate; CI, confidence interval; cNRI, continuous net reclassification improvement; IDI, integrated discrimination improvement.

## Discussion

In the present study, the prevalence of SF was 61% in hospitalized older HF patients. The composite event rates after discharge were higher in patients with SF than in patients without SF and the presence of SF was an independent predictor of the composite event rate. These findings were consistent with the results of an earlier study (13). Additionally, among five questions, loss of perceived social role was the sole predictor of composite events and gave an additive impact to future event prediction by established markers. This is the first study showing a close association between loss of perceived social role and poor clinical outcome in older HF patients.

### Association between social frailty and clinical outcome in older heart failure patients

Social determinants of health (SDoH), including economic, social, environmental, and psychosocial factors that influence health, have been shown to play a significant role in morbidity and mortality of various diseases including cardiovascular disease (CVD) (28). The importance of SDoH in the field of medical care has been re-recognized: SDoH have considerable effects on novel coronavirus disease 2019 (COVID-19) mortality and morbidity (29). Several factors of SDoH such as self-reported perceived stress, subjective social status, job strain, perceived discrimination, and loneliness have been shown to be implicated in increased mortality and morbidity (28), one of which is SF. Evidence has shown that presence of frailty evaluated by checklists including social aspects, e.g., the Kihon Checklist and the Kaigo-Yobo Checklist, predicts morbidity and mortality in community-dwelling older people (30). Although there is no standardized method for evaluating SF specifically, a candidate method is Makizako’s 5 questions (14). Five questions for defining SF were selected on the basis of the associations between questions regarding social issues and incident disability during a 2-year follow-up period in community-dwelling older people (14). When people with negative responses to 2 or more questions were considered to have SF, the presence of SF predicted the development of disability independently of multiple risk factors of disability. In addition, results of two studies including this study indicated that SF is an independent predictor of mortality and HF hospitalization in older hospitalized HF patients (13). Thus, although the usefulness of SF assessed by Makizako’s 5 questions for prediction of clinical outcomes has been validated by two studies, further analyses are needed to determine the best SF definition for predicting future decline in ADL, HF rehospitalization, and mortality in older HF patients.

Previous research has shown that SF status, including low social activity, low social role, and poor social relationships, causes the onset and progression of physical frailty (16), poor physical performance (31), and malnutrition (32), resulting in disabilities (14, 33, 34), and mortality (32, 33) in community-dwelling older adults. Close relationships between SF and physical frailty, physical performance, dietary condition, and cognitive function can all exacerbate poor health state and lead to unfavorable consequences. Even after adjusting for age and gender, our cross-sectional analyses revealed that HF patients in the SF group had a significantly higher prevalence of physical frailty and lower status of physical function, nutrition, and cognitive performance than those in the non-SF group (Supplementary Table 4), which are consistent with earlier findings in community-dwelling older adults (10, 35). However, the cause-and-effect relationship among the frailty syndrome remains to be elucidated. HF causes exercise intolerance, contributing to development of SF through social isolation and perceived social roles due to fewer opportunities to engage with people. Loss of social activity can lead to exaggeration of poor physical and mental status. Indeed, the results of a previous study in community-dwelling older people showed that the presence of SF may be an antecedent event of physical and psychological frailty. This hypothesis was supported by a close association between SF and poor clinical outcome in HF patients: SF was linked with the occurrence of composite events in HF patients independent of physical and psychological factors (Table 2). Taken together, development of SF may be a primary mechanism in the progression of frailty syndrome in HF patients. Nevertheless, more research is needed to unveil the complex relationship between frailty syndrome and adverse outcomes in HF patients.

### Perceived social role and clinical outcome in heart failure patients: Clinical implications

On the other hand, which questions for defining SF have an untoward impact on prognosis have not been elucidated. There are several factors included in SF, i.e., social isolation, loneliness, low level of social activity, lack of social support, and loss of perceived social role, which are intertwined with each other and are not easily discriminated. However, among Makizako’s 5 questions, loss of perceived social role had the greatest impact on future adverse events and had additive predictive value when added to the model including pre-existing prognostic markers in the present study. In community-dwelling older people, such relationships between low perceived usefulness and unfavorable clinical outcomes such as disability, progression of organic diseases, and mortality have been shown (14, 36, 37). Those who believe themselves not to be helpful have less social connections, worse self-efficacy and control, less social support, and lower resilience than those who believe themselves to be useful (36, 37). Furthermore, a poor sense of usefulness has been shown to lead to fewer social activities and health-seeking behaviors, contributing to the development or exaggeration of health problems including disability (14, 38–40). Although there were no data focusing on the role of a low social role in older HF patients, results of previous studies in HF patients showed that social isolation—defined as an objective lack of or disengagement from social ties, institutional connections, or community participation (41)—is associated with adverse outcomes (42–46). Importantly, social isolation was shown to be associated with a 55% greater risk of hospital readmission for HF in a recent meta-analysis in which 42% Asian data out of the whole sample were included. In addition, only loss of perceived social role was associated with all indexes of physical and psychological frailty, i.e., Barthel index score, gait speed, handgrip strength, and Mini-cog score, in the present study (Supplementary Table 4). Importantly, having a greater number of valued social roles is associated with less prevalence of depression (47), which is frequently found in HF patients (48). Taken together, loss of perceived social role is a critical component of SF in treatment of older patients with HF. Nevertheless, prospective interventional studies such as participation in domestic tasks and social activities such as engagement in meaningful volunteer activities are needed to demonstrate whether loss of perceived social role is an antecedent event of frailty syndrome and adverse events in older HF patients.

### Study strengths and limitations

This study has several strengths. First, there were no missing data for assessment of SF by using Makizako’s five questions in the entire cohort, and that helped us to analyze the relative importance of each question in prediction of clinical outcome in HF patients. Second, the post-discharge follow-up period in this study was longer (up to 3 years) than the periods in the previous studies (from 90 days to 1 year). (13, 44) This enabled us to examine the long-term impact of SF on adverse clinical outcomes in elderly HF patients. Finally, we performed multiple imputations by a chained equation to account for potential selection bias and inefficient analysis due to missing data.

It is also important to note that this study has several limitations. First, because this study was a retrospective observational study with a small number of patients and patients with missing data in a single center, there could have been selection bias in study subjects even after multiple imputation techniques. Furthermore, our findings may not be applicable to patients who cannot walk even with assistive aids. Second, while there were no differences in the severity of NYHA functional class, frequency of HF phenotype based on LVEF, and medication use between SF and non-SF groups (Table 1), the *post hoc* analyses were performed to demonstrate the prognostic impact of SF among subgroups of patients. As shown in Supplementary Figure 1, prognostic impacts of SF were consistent across subgroups except for HF phenotype (i.e., HFrEF vs. non-HFrEF), indicating prognostic impacts of SF are independent of HF severity and therapies. Although the prognostic impact of SF was independent of HFrEF in the fully adjusted multivariate Cox proportional hazard models (Table 2), it may be prominent in the non-HFrEF group. Future studies with a large sample size are needed to demonstrate the mechanism of prognostic significance of SF in the non-HFrEF patients. Third, the instrument utilized in this study has not been validated in HF patients. However, since the test items used in this study are not costly or time-consuming and are helpful in determining prognosis, future studies should seek validation from an outside institution. Furthermore, we acknowledge that perceived usefulness was assessed using only a single question, which may have resulted in measurement bias. Therefore, we recommend more research to back up these findings with more sophisticated and multidomain measures that better capture the complex nature of self-perceptions of usefulness. Fourth, changes the status of SF and feelings of the social role changed over time were not examined in this study. Previous studies showed that a decrease in feelings of usefulness over time was associated with a higher risk for mortality in older adults (36, 49, 50). Further research is needed to determine the prognostic significance of changes in the severity of SF and the feelings of usefulness in HF patients over time. Finally, only Japanese patients were included in this study. Considering the racial, regional and age differences in cardiovascular outcomes and the etiology of HF, this study’s findings may not be applicable to HF patients in other countries. Importantly, the most frequent etiology of HF was VHD in the present study. The impacts of HF etiology on the significance of SF in the occurrence of adverse events in HF patients were separately analyzed in the future study.

## Conclusion

In addition to SF, perceived social role was associated with increased composite event risk in older HF patients and provided further predictive information in older HF patients, suggesting the importance of the social role in the management of older HF patients.

## Data availability statement

The original contributions presented in this study are included in the article/Supplementary material, further inquiries can be directed to the corresponding author.

## Ethics statement

The studies involving human participants were reviewed and approved by the Clinical Investigation Ethics Committee of Sapporo Medical University Hospital. Written informed consent was not provided because the opt-out method was applied to obtain consent for this study.

## Author contributions

AW, SK, AH, TY, and MF contributed to the conception and design of the work. SK, AW, RNa, RNu, SH, KY, YF, KO, HK, TI, MK, AH, TY, and MF contributed to the acquisition, analysis, and interpretation of the data for the current study. AW, SK, and TY performed the statistical analyses. SK, AW, MK, AH, and TY drafted the manuscript. MK, TY, and MF critically revised the manuscript. All authors provided the final approval and agreed to be accountable for all aspects of the work ensuring integrity and accuracy.

## Footnotes

1
https://www.R-project.org/

## Abbreviations

ADL: activities of daily living
CCI: Charlson comorbidity index
CKD: chronic kidney disease
cNRI: continuous net reclassification improvement
eGFRcys: cystatin C-based glomerular filtration rate
HF: heart failure
HFrEF: heart failure with reduced ejection fraction
HFpEF: heart failure with preserved ejection fraction
IDI: integrated discrimination improvement
IQR: interquartile range
LVEF: left ventricular ejection fraction
MNA-SF: mini nutritional assessment short form
NT-proBNP: N-terminal pro-brain natriuretic peptide
SF: social frailty.

## References

[1] Savarese G Lund LH. Global public health burden of heart failure. *Card Fail Rev.* (2017) 3:7–11. 10.15420/cfr.2016:25:2 28785469 PMC5494150

[2] Ambrosy AP Fonarow GC Butler J Chioncel O Greene SJ Vaduganathan M The global health and economic burden of hospitalizations for heart failure. *J Am Coll Cardiol.* (2014) 63:1123–33. 10.1016/j.jacc.2013.11.053 24491689

[3] Constantinou P Pelletier-Fleury N Olié V Gastaldi-Ménager C JuillÈre Y Tuppin P. Patient stratification for risk of readmission due to heart failure by using nationwide administrative data. *J Card Fail.* (2021) 27:266–76. 10.1016/j.cardfail.2020.07.018 32801005

[4] Tsutsui H Isobe M Ito H Ito H Okumura K Ono M JCS 2017/JHFS 2017 guideline on diagnosis and treatment of acute and chronic heart failure —digest version —. *Circ J.* (2019) 83:2084–184. 10.1253/circj.cj-19-0342 31511439

[5] Shiraishi Y Kohsaka S Sato N Takano T Kitai T Yoshikawa T 9-Year trend in the management of acute heart failure in Japan: a report from the national consortium of acute heart failure registries. *J Am Heart Assoc.* (2018) 7:e008687. 10.1161/jaha.118.008687 30371201 PMC6222932

[6] Katano S Hashimoto A Ohori K Watanabe A Honma R Yanase R Nutritional status and energy intake as predictors of functional status after cardiac rehabilitation in elderly inpatients with heart failure —a retrospective cohort study —. *Circ J.* (2018) 82:1584–91. 10.1253/circj.cj-17-1202 29628459

[7] Palazzuoli A Ruocco G Gronda E. Noncardiac comorbidity clustering in heart failure: an overlooked aspect with potential therapeutic door. *Heart Fail Rev.* (2022) 27:767–78. 10.1007/s10741-020-09972-6 32382883

[8] Rockwood K. What would make a definition of frailty successful? *Age Ageing.* (2005) 34:432–4. 10.1093/ageing/afi146 16107450

[9] Fried LP Tangen CM Walston J Newman AB Hirsch C Gottdiener J Frailty in older adults: evidence for a phenotype. *J Gerontol A Biol Sci Med Sci.* (2001) 56:M146–57. 10.1093/gerona/56.3.m146 11253156

[10] Tsutsumimoto K Doi T Makizako H Hotta R Nakakubo S Makino K Association of social frailty with both cognitive and physical deficits among older people. *J Am Med Dir Assoc.* (2017) 18:603–7. 10.1016/j.jamda.2017.02.004 28411094

[11] Kitzman DW Whellan DJ Duncan P Pastva AM Mentz RJ Reeves GR Physical rehabilitation for older patients hospitalized for heart failure. *N Engl J Med.* (2021) 385:203–16. 10.1056/nejmoa2026141 33999544 PMC8353658

[12] Kamiya K Sato Y Takahashi T Tsuchihashi-Makaya M Kotooka N Ikegame T Multidisciplinary cardiac rehabilitation and long-term prognosis in patients with heart failure. *Circ Heart Fail.* (2020) 13:e006798. 10.1161/circheartfailure.119.006798 32986957

[13] Jujo K Kagiyama N Saito K Kamiya K Saito H Ogasahara Y Impact of social frailty in hospitalized elderly patients with heart failure: a FRAGILE-HF registry subanalysis. *J Am Heart Assoc.* (2020) 10:e019954. 10.1161/jaha.120.019954 34472374 PMC8649263

[14] Makizako H Shimada H Tsutsumimoto K Lee S Doi T Nakakubo S Social frailty in community-dwelling older adults as a risk factor for disability. *J Am Med Dir Assoc.* (2015) 16:1003.e7–11. 10.1016/j.jamda.2015.08.023 26482055

[15] Makizako H Kubozono T Kiyama R Takenaka T Kuwahata S Tabira T Associations of social frailty with loss of muscle mass and muscle weakness among community-dwelling older adults. *Geriatr Gerontol Int.* (2019) 19:76–80. 10.1111/ggi.13571 30575241

[16] Makizako H Shimada H Doi T Tsutsumimoto K Hotta R Nakakubo S Social frailty leads to the development of physical frailty among physically non-frail adults: a four-year follow-up longitudinal cohort study. *Int J Environ Res Public Health.* (2018) 15:490. 10.3390/ijerph15030490 29534470 PMC5877035

[17] Horio M Imai E Yasuda Y Watanabe T Matsuo S Collaborators Developing the Japanese Equation for Estimated GFR. GFR estimation using standardized serum cystatin C in Japan. *Am J Kidney Dis.* (2013) 61:197–203. 10.1053/j.ajkd.2012.07.007 22892396

[18] Kawashiro N Kasanuki H Ogawa H Matsuda N Hagiwara N Heart Institute of Japan–Department of Cardiology (HIJC) Investigators. Clinical characteristics and outcome of hospitalized patients with congestive heart failure. *Circ J.* (2008) 72:2015–20. 10.1253/circj.cj-08-0323 18931450

[19] Katano S Yano T Ohori K Kouzu H Nagaoka R Honma S Barthel index score predicts mortality in elderly heart failure — a goal of comprehensive cardiac rehabilitation. *Circ J.* (2021) 86:70–8. 10.1253/circj.cj-21-0584 34544962

[20] Katano S Yano T Kouzu H Ohori K Shimomura K Honma S Energy intake during hospital stay predicts all-cause mortality after discharge independently of nutritional status in elderly heart failure patients. *Clin Res Cardiol.* (2021) 110:1202–20. 10.1007/s00392-020-01774-y 33399954 PMC8318973

[21] GroupMIKaiserMJBauerJMRamschCUterWGuigozY Validation of the mini nutritional assessment short-form (MNA^®^ -SF): a practical tool for identification of nutritional status. *J Nutr Health Aging.* (2009) 13:782. 10.1007/s12603-009-0214-7 19812868

[22] Charlson ME Pompei P Ales KL MacKenzie CR. A new method of classifying prognostic comorbidity in longitudinal studies: development and validation. *J Chron Dis.* (1987) 40:373–83. (87)90171-8 10.1016/0021-9681 3558716

[23] Borson S Scanlan J Brush M Vitaliano P Dokmak A. The mini-cog: a cognitive “vital signs” measure for dementia screening in multi-lingual elderly. *Int J Geriatr Psychiatry.* (2000) 15:1021–7. (200011)15:113.0.co;2-6 10.1002/1099-1166 11113982

[24] Satake S Arai H. The revised Japanese version of the cardiovascular health study criteria (revised J-CHS criteria). *Geriatr Gerontol Int.* (2020) 20:992–3. 10.1111/ggi.14005 33003255

[25] Pencina MJ D’Agostino RB D’Agostino RB Vasan RS. Evaluating the added predictive ability of a new marker: from area under the ROC curve to reclassification and beyond. *Stat Med.* (2008) 27:157–72. 10.1002/sim.2929 17569110

[26] Pencina MJ D’Agostino RB Steyerberg EW. Extensions of net reclassification improvement calculations to measure usefulness of new biomarkers. *Stat Med.* (2011) 30:11–21. 10.1002/sim.4085 21204120 PMC3341973

[27] Austin PC White IR Lee DS van Buuren S. Missing data in clinical research: a tutorial on multiple imputation. *Can J Cardiol.* (2020) 37:1322–31. 10.1016/j.cjca.2020.11.010 33276049 PMC8499698

[28] Powell-Wiley TM Baumer Y Baah FO Baez AS Farmer N Mahlobo CT Social determinants of cardiovascular disease. *Circ Res.* (2022) 130:782–99. 10.1161/circresaha.121.3198110 35239404 PMC8893132

[29] Abrams EM Szefler SJ. COVID-19 and the impact of social determinants of health. *Lancet Respir Med.* (2020) 8:659–61. 10.1016/s2213-2600(20)30234-4 32437646 PMC7234789

[30] Kojima G Taniguchi Y Kitamura A Shinkai S. Are the kihon checklist and the kaigo-yobo checklist compatible with the frailty index? *J Am Med Dir Assoc.* (2018) 19:797–800.e2. 10.1016/j.jamda.2018.05.012 29980481

[31] Tanaka T Son B Lyu W Iijima K. Impact of social engagement on the development of sarcopenia among community-dwelling older adults: a Kashiwa cohort study. *Geriatr Gerontol Int.* (2022) 22:384–91. 10.1111/ggi.14372 35322539

[32] Huang CH Okada K Matsushita E Uno C Satake S Martins BA Sex-specific association between social frailty and diet quality, diet quantity, and nutrition in community-dwelling elderly. *Nutrients.* (2020) 12:2845. 10.3390/nu12092845 32957506 PMC7551288

[33] Yamada M Arai H. Social frailty predicts incident disability and mortality among community-dwelling Japanese older adults. *J Am Med Dir Assoc.* (2018) 19:1099–103. 10.1016/j.jamda.2018.09.013 30471801

[34] Teo N Gao Q Nyunt MSZ Wee SL Ng TP. Social frailty and functional disability: findings from the Singapore longitudinal ageing studies. *J Am Med Dir Assoc.* (2017) 18:637.e13–637.e19. 10.1016/j.jamda.2017.04.015 28648903

[35] MaLSunFTangZ. Social frailty is associated with physical functioning, cognition, and depression, and predicts mortality. *J Nutr Health Aging.* (2018) 22:989–95. 10.1007/s12603-018-1054-0 30272104

[36] Gruenewald TL Karlamangla AS Greendale GA Singer BH Seeman TE. Increased mortality risk in older adults with persistently low or declining feelings of usefulness to others. *J Aging Health.* (2009) 21:398–425. 10.1177/0898264308329023 19104034 PMC2747376

[37] Gruenewald TL Karlamangla AS Greendale GA Singer BH Seeman TE. Feelings of usefulness to others, disability, and mortality in older adults: the macarthur study of successful aging. *J Gerontol B Psychol Sci Soc Sci.* (2007) 62:28–37. 10.1093/geronb/62.1.p28 17284554

[38] Wurm S Benyamini Y. Optimism buffers the detrimental effect of negative self-perceptions of ageing on physical and mental health. *Psychol Health.* (2014) 29:832–48. 10.1080/08870446.2014.891737 24527737

[39] Wurm S Warner LM Ziegelmann JP Wolff JK Schüz B. How do negative self-perceptions of aging become a self-fulfilling prophecy? *Psychol Aging.* (2013) 28:1088–97. 10.1037/a0032845 24128074

[40] Sarkisian CA Hays RD Mangione CM. Do older adults expect to age successfully? the association between expectations regarding aging and beliefs regarding healthcare seeking among older adults. *J Am Geriatr Soc.* (2002) 50:1837–43. 10.1046/j.1532-5415.2002.50513.x 12410903

[41] Seeman TE. Social ties and health: the benefits of social integration. *Ann Epidemiol.* (1996) 6:442–51. (96)00095-6 10.1016/s1047-2797 8915476

[42] Rodríguez-Artalejo F Guallar-Castillón P Herrera MC Otero CM Chiva MO Ochoa CC Social network as a predictor of hospital readmission and mortality among older patients with heart failure. *J Card Fail.* (2006) 12:621–7. 10.1016/j.cardfail.2006.06.471 17045181

[43] Gorji MAH Fatahian A Farsavian A. The impact of perceived and objective social isolation on hospital readmission in patients with heart failure: a systematic review and meta-analysis of observational studies. *Gen Hosp Psychiatry.* (2019) 60:27–36. 10.1016/j.genhosppsych.2019.07.002 31310898

[44] Saito H Kagiyama N Nagano N Matsumoto K Yoshioka K Endo Y Social isolation is associated with 90-day rehospitalization due to heart failure. *Eur J Cardiovasc Nurs.* (2018) 18:16–20. 10.1177/1474515118800113 30251884

[45] Tsuchihashi-Makaya M Kato N Chishaki A Takeshita A Tsutsui H. Anxiety and poor social support are independently associated with adverse outcomes in patients with mild heart failure. *Circ J.* (2009) 73:280–7. 10.1253/circj.cj-08-0625 19096191

[46] Sokoreli I Pauws SC Steyerberg EW de Vries G Riistama JM Tesanovic A Prognostic value of psychosocial factors for first and recurrent hospitalizations and mortality in heart failure patients: insights from the OPERA-HF study. *Eur J Heart Fail.* (2018) 20:689–96. 10.1002/ejhf.1112 29314447

[47] Hunt MG Stein CH. Valued social roles and measuring mental health recovery: examining the structure of the tapestry. *Psychiatr Rehabil J.* (2012) 35:441–6. 10.1037/h0094577 23276237 PMC4037131

[48] Sbolli M Fiuzat M Cani D O’Connor CM. Depression and heart failure: the lonely comorbidity. *Eur J Heart Fail.* (2020) 22:2007–17. 10.1002/ejhf.1865 32468714

[49] Zhao Y Dupre ME Qiu L Gu D. Changes in perceived uselessness and risks for mortality: evidence from a national sample of older adults in China. *BMC Public Health.* (2017) 17:561. 10.1186/s12889-017-4479-1 28599631 PMC5466746

[50] Curzio O Bernacca E Bianchi B Rossi G. Feelings of uselessness and 3-year mortality in an Italian community older people: the role of the functional status. *Psychogeriatrics.* (2017) 17:300–9. 10.1111/psyg.12238 28130890

