# Supplementary material for: Loss of perceived social role, an index of social frailty, is an independent predictor of future adverse events in hospitalized patients with heart failure
Source: Front Cardiovasc Med. 2022 Dec 20;9:1051570. doi: 10.3389/fcvm.2022.1051570 (PMC9807608; doi:10.3389/fcvm.2022.1051570)
Supplement: Supplementary file 1 [file Data_Sheet_1.docx]

**Supplementary Table 1** Makizako’s five questions and its scoring method

| Makizako’s 5 items | yes | no |
| --- | --- | --- |
| Do you go out less frequently compared with last year? | 1 | 0 |
| Do you sometimes visit your friends? | 0 | 1 |
| Do you feel you are helpful toward friends or family? | 0 | 1 |
| Do you live alone? | 1 | 0 |
| Do you talk with someone every day? | 0 | 1 |

**Supplementary Table 2**. Comparison of baseline characteristics in patients with complete data and those with missing data

|  | Missing | | Overall | |  | Patients with complete data sets | |  | Patients with missing data sets | | p value |
| --- | --- | --- | --- | --- | --- | --- | --- | --- | --- | --- | --- |
|  | n (%) | | n = 310 | |  | n = 259 | |  | n = 51 | |  |
| Age, *years* |  |  | 79 | (72, 84) |  | 79 | (72, 85) |  | 78 | (71, 83) | 0.29 |
| Women, *n (%)* |  |  | 143 | (46) |  | 134 | (52) |  | 33 | (65) | 0.09 |
| Height, *cm* |  |  | 157 | ± 10 |  | 157 | ± 10 |  | 159 | ± 9 | 0.22 |
| Body weight, *kg* |  |  | 55.4 | (47.0, 61.6) |  | 55.5 | (46.3, 62.1) |  | 54.0 | (48.3, 60.5) | 0.95 |
| BMI, *kg/m^2^* |  |  | 21.8 | (19.5, 23.9) |  | 22.1 | (19.6, 24.0) |  | 21.5 | (19.3, 23.6) | 0.33 |
| Systolic blood pressure, *mmHg* |  |  | 117 | (105, 131) |  | 118 | (107,132) |  | 110 | (101, 124) | 0.02 |
| NYHA functional class, *n (%)* |  |  |  |  |  |  |  |  |  |  |  |
| Ⅰ |  |  | 16 | (5) |  | 14 | (5) |  | 2 | (4) | 0.65 |
| Ⅱ |  |  | 183 | (59) |  | 155 | (60) |  | 28 | (55) |  |
| Ⅲ |  |  | 111 | (36) |  | 90 | (35) |  | 21 | (41) |  |
| LVEF, *%* |  |  | 51.1 | (35.1, 63.5) |  | 52.3 | (37.3, 63.8) |  | 46.5 | (27.4, 62.4) | 0.03 |
| HFrEF, *n (%)* |  |  | 94 | (30) |  | 72 | (28) |  | 22 | (44) | 0.07 |
| HFpEF, *n (%)* |  |  | 167 | (54) |  | 146 | (56) |  | 21 | (42) |  |
| Prior hospital admission due to HF, *n (%)* |  |  | 129 | (42) |  | 99 | (38) |  | 30 | (59) | < 0.01 |
| Etiology, *n (%)* |  |  |  |  |  |  |  |  |  |  |  |
| Cardiomyopathy |  |  | 77 | (25) |  | 63 | (24) |  | 14 | (27) | 0.49 |
| Valvular heart disease |  |  | 130 | (42) |  | 110 | (42) |  | 20 | (39) |  |
| Ischemic |  |  | 50 | (16) |  | 39 | (15) |  | 11 | (22) |  |
| Comorbidity, *n (%)* |  |  |  |  |  |  |  |  |  |  |  |
| Hypertension |  |  | 217 | (70) |  | 183 | (71) |  | 34 | (67) | 0.57 |
| Dyslipidemia |  |  | 188 | (61) |  | 155 | (60) |  | 33 | (65) | 0.52 |
| Diabetes mellitus |  |  | 127 | (41) |  | 101 | (39) |  | 26 | (51) | 0.11 |
| Atrial fibrillation |  |  | 126 | (41) |  | 106 | (41) |  | 20 | (39) | 0.82 |
| Cancer |  |  | 84 | (27) |  | 69 | (27) |  | 15 | (29) | 0.68 |
| Charlson comorbidity index, *points* |  |  | 3 | (2, 4) |  | 3 | (2, 4) |  | 3 | (2, 5) | 0.19 |
| 10-m gait speed, *m/sec* | 6 | (2) | 0.831 | ± 0.276 |  | 0.843 | ± 0.281 |  | 0.763 | ± 0.239 | 0.049 |
| Hand grip strength, *kg* | 23 | (7) | 21.1 | (15.1, 27.9) |  | 21.0 | (14.8, 28.1) |  | 22.8 | (16.7, 27.9) | 0.61 |
| Barthel Index, *points* |  |  | 90 | (80, 95) |  | 90 | (80, 95) |  | 85 | (70, 95) | 0.04 |
| MNA-SF score |  |  | 9 | (7, 11) |  | 9 | (7, 11) |  | 8 | (6, 10) | 0.27 |
| Laboratory data |  |  |  |  |  |  |  |  |  |  |  |
| NT-proBNP, *pg/mL* |  |  | 1192 | (507, 2749) |  | 1195 | (508, 2639) |  | 1067 | (496, 2877) | 0.51 |
| Albumin, *g/dL* |  |  | 3.5 | (3.2, 3.7) |  | 3.5 | (3.3, 3.7) |  | 3.5 | (3.1, 3.7) | 0.37 |
| Hemoglobin, *g/dL* |  |  | 11.4 | (10.4, 12.9) |  | 11.4 | (10.5, 12.9) |  | 11.2 | (9.9, 13.1) | 0.17 |
| Cystatin C | 20 | (6) | 1.24 | (1.04, 1.68) |  | 1.23 | (1.04, 1.65) |  | 1.31 | (1.11, 2.15) | 0.09 |
| eGFRcys, *mL/min/1.73m^2^* | 20 | (6) | 50.3 | (34.9, 64.8) |  | 51.2 | (36.3, 64.9) |  | 43.8 | (25.8, 60.8) | 0.13 |
| Medication, *n (%)* |  |  |  |  |  |  |  |  |  |  |  |
| β blocker |  |  | 193 | (62) |  | 161 | (62) |  | 30 | (60) | 0.75 |
| ACE-I or ARB |  |  | 170 | (55) |  | 142 | (55) |  | 27 | (54) | 0.89 |
| MRA |  |  | 139 | (45) |  | 114 | (44) |  | 24 | (48) | 0.62 |
| Loop diuretics |  |  | 195 | (63) |  | 159 | (62) |  | 35 | (70) | 0.26 |
| Physical frailty, *n (%)* | 27 | (9) | 156 | (55) |  | 144 | (56) |  | 12 | (50) | 0.60 |
| Social frailty, *n (%)* |  |  | 188 | (61) |  | 159 | (61) |  | 29 | (57) | 0.55 |
| Makizako’s social frailty score, *points* |  |  | 2 | (1, 3) |  | 2 | (1, 3) |  | 2 | (1, 3) | 0.62 |
| Do you go out less frrequently compared with last year? (yes), *n (%)* |  |  | 211 | (68) |  | 181 | (70) |  | 30 | (59) | 0.12 |
| Do you sometimes visit your friends? (no), *n (%)* |  |  | 202 | (65) |  | 167 | (64) |  | 35 | (69) | 0.57 |
| Do your feel you are helpful to friends or family? (n0), *n (%)* |  |  | 81 | (26) |  | 66 | (25) |  | 15 | (29) | 0.56 |
| Do you live alone? (yes), *n (%)* |  |  | 64 | (21) |  | 54 | (21) |  | 10 | (20) | 0.84 |
| Do you talk with someone every day? (no), *n (%)* |  |  | 36 | (12) |  | 32 | (12) |  | 4 | (8) | 0.36 |
| Cohabitants, yes, *n (%)* |  |  | 246 | (79) |  | 205 | (79) |  | 41 | (80) | 0.84 |
| Long-term care insurance, *n (%)* |  |  | 129 | (42) |  | 107 | (41) |  | 22 | (43) | 0.81 |
| Cognitive function |  |  |  |  |  |  |  |  |  |  |  |
| Mini-cog score, *point* | 7 | (2) | 5 | (4, 5) |  | 5 | (4, 5) |  | 5 | (4, 5) | 0.15 |
| Cognitive impairement, *n (%)* | 7 | (2) | 31 | (10) |  | 28 | (11) |  | 3 | (7) | 0.42 |

Data are presented as mean ± standard deviation of the mean, median (interquartile range, 25^th^, 75^th^ percentile), or number (with percentage). n, number of patients for whom the parameter was available. Abbreviations: BMI, body mass index; NYHA, New York Heart Association; LVEF, left ventricular ejection fraction; HFrEF, heart failure with reduced ejection fraction; HFpEF, heart failure with preserved ejection fraction; HF, heart failure; MNA-SF, mini nutritional assessment short form; NT-proBNP, N-terminal pro B-type natriuretic peptide; eGFRcys*,* cystatin C-based estimated glomerular filtration rate; ACE-I, angiotensin-converting enzyme inhibitor; ARB, angiotensin receptor blocker; MRA, mineralocorticoid receptor antagonist.

**Supplementary Table 3**. Cox proportional hazard model to predict the composite event in older HF patients with/without a negative response to each subitem of the questionnaire.

|  | Multivariate model  (Multiple imputed case) | | |  | Multivariate model  (Complete case, n = 259) | | |
| --- | --- | --- | --- | --- | --- | --- | --- |
| Makizako’s 5 items | HR | (95% CI) | p value |  | HR | (95% CI) | p value |
| Do you go out less frequently compared with last year? (yes) | 1.01 | (0.57, 1.79) | 0.97 |  | 1.04 | (0.59, 1.84) | 0.88 |
| Do you sometimes visit your friends? (no) | 1.86 | (0.998, 3.48) | 0.06 |  | 1.67 | (0.91, 3.08) | 0.10 |
| Do you feel your are helpful toward friends or family? (no) | 2.28 | (1.36, 3.82) | < 0.01 |  | 2.07 | (1.23, 3.47) | < 0.01 |
| Do you live alone? (yes) | 1.13 | (0.68, 2.11) | 0.70 |  | 1.14 | (0.61, 2.14) | 0.67 |
| Do you talk with someone every day? (no) | 1.56 | (0.77, 3.16) | 0.22 |  | 1.90 | (0.97, 3.74) | 0.06 |

Multivariate model was adjusted for age, sex, prior hospital admission due to HF, HFrEF, log NT-proBNP, eGFRcys, Charlson comorbidity index, and physical frailty. Abbreviations: HF, heart failure; HR, hazard ratio; CI, confidence interval; HfrEF, heart failure with reduced ejection fraction; log NT-proBNP, logarithmic N-terminal pro B-type natriuretic peptide; eGFRcys, cystatine C-based estimated glomerular filtration rate.

**Supplementary Table 4**. Comparisons of physical and cognitive functions, and nutritional status

|  |  |  | Physical frailty | | |  | Barthel Index score (points) | | |  | Gait speed (m/sec) | | |  | Handgrip strength (kg) | | |  | Mini-cog score (points) | | |
| --- | --- | --- | --- | --- | --- | --- | --- | --- | --- | --- | --- | --- | --- | --- | --- | --- | --- | --- | --- | --- | --- |
|  |  | n | OR | (95% CI) | p |  | LSM | (95% CI) | p |  | LSM | (95% CI) | p |  | LSM | (95% CI) | p |  | LSM | (95% CI) | p |
| Social frailty | Yes | 188 | 1.10 | (0.98, 1.23) | 0.10 |  | 83 | (81, 85) | 0.02 |  | 0.798 | (0.762, 0.834) | 0.04 |  | 21.0 | (20.1, 21.9) | 0.03 |  | 4.2 | (4.0, 4.3) | 0.16 |
|  | No | 122 | 1.00 | (Reference) |  |  | 87 | (84, 89) |  |  | 0.860 | (0.814, 0.905) |  |  | 22.5 | (21.5, 23.6) |  |  | 4.3 | (4.1, 4.6) |  |
|  |  |  |  |  |  |  |  |  |  |  |  |  |  |  |  |  |  |  |  |  |  |
| Do you go out less frequently compared with last year? | Yes | 211 | 1.08 | (0.96, 1.21) | 0.21 |  | 84 | (82, 86) | 0.42 |  | 0.812 | (0.778, 0.846) | 0.30 |  | 21.3 | (20.5,22.1) | 0.13 |  | 4.3 | (4.1, 4.4) | 0.37 |
|  | No | 99 | 1.00 | (Reference) |  |  | 86 | (83, 88) |  |  | 0.844 | (0.794, 0.893) |  |  | 22.3 | (21.2, 23.5) |  |  | 4.1 | (3.9, 4.4) |  |
|  |  |  |  |  |  |  |  |  |  |  |  |  |  |  |  |  |  |  |  |  |  |
| Do you sometimes visit your friend? | Yes | 108 | 1.00 | (Reference) | 0.11 |  | 84 | (82, 86) | 0.42 |  | 0.881 | (0.834, 0.928) | < 0.01 |  | 22.8 | (21.7, 24.0) | < 0.01 |  | 4.4 | (4.2, 4.7) | 0.02 |
|  | No | 202 | 1.10 | (0.98, 1.23) |  |  | 86 | (83, 88) |  |  | 0.790 | (0.755, 0.824) |  |  | 20.9 | (20.1, 21.8) |  |  | 4.1 | (4.0, 4.3) |  |
|  |  |  |  |  |  |  |  |  |  |  |  |  |  |  |  |  |  |  |  |  |  |
| Do you feel you are helpful to friends or family? | Yes | 229 | 1.00 | (Reference) | < 0.01 |  | 87 | (85, 89) | < 0.01 |  | 0.864 | (0.832, 0.895) | < 0.01 |  | 22.5 | (21.8, 23.3) | < 0.01 |  | 4.3 | (4.2, 4.5) | 0.01 |
|  | No | 81 | 1.25 | (1.10, 1.42) |  |  | 78 | (75, 81) |  |  | 0.706 | (0.652, 0.759) |  |  | 19.0 | (17.7, 20.3) |  |  | 4.0 | (3.7, 4.2) |  |
|  |  |  |  |  |  |  |  |  |  |  |  |  |  |  |  |  |  |  |  |  |  |
| Do you Live alone? | Yes | 64 | 1.00 | (0.87, 1.15) | 0.99 |  | 84 | (81, 88) | 0.81 |  | 0.873 | (0.812, 0.935) | 0.07 |  | 22.2 | (20.7, 23.7) | 0.38 |  | 4.3 | (4.0, 4.6) | 0.44 |
|  | No | 246 | 1.00 | (Reference) |  |  | 85 | (83, 86) |  |  | 0.808 | (0.776, 0.840) |  |  | 21.4 | (20.7, 22.2) |  |  | 4.2 | (4.1, 4.4) |  |
|  |  |  |  |  |  |  |  |  |  |  |  |  |  |  |  |  |  |  |  |  |  |
| Do you talk with someone everyday? | Yes | 274 | 1.00 | (Reference) | 0.36 |  | 85 | (83, 86) | 0.76 |  | 0.818 | (0.788, 0.848) | 0.49 |  | 21.6 | (20.9, 22.3) | 0.88 |  | 4.2 | (4.1, 4.4) | 0.53 |
|  | No | 36 | 0.92 | (0.78, 1.09) |  |  | 85 | (81, 90) |  |  | 0.849 | (0.767, 0.931) |  |  | 21.5 | (19.5, 23.4) |  |  | 4.3 | (4.0, 4.1) |  |

Models were constructed using logistic regression analysis and analysis of covariance and were adjusted for age and sex. Abbreviations: OR, odds ratio; CI, confidence interval; LSM, least square mean.

**Supplementary Table 4**. Comparisons of physical and cognitive functions, and nutritional status (continued)

|  |  |  | BMI | | |  | MNA-SF score (points) | | |  | Albumin (g/dL) | | |  | Hemoglobin (g/dL) | | |
| --- | --- | --- | --- | --- | --- | --- | --- | --- | --- | --- | --- | --- | --- | --- | --- | --- | --- |
|  |  | n | OR | (95% CI) | p |  | LSM | (95% CI) | p |  | LSM | (95% CI) | p |  | LSM | (95% CI) | p |
| Social frailty | Yes | 188 | 1.10 | (0.98, 1.23) | 0.10 |  | 83 | (81, 85) | 0.02 |  | 0.798 | (0.762, 0.834) | 0.04 |  | 21.0 | (20.1, 21.9) | 0.03 |
|  | No | 122 | 1.00 | (Reference) |  |  | 87 | (84, 89) |  |  | 0.860 | (0.814, 0.905) |  |  | 22.5 | (21.5, 23.6) |  |
|  |  |  |  |  |  |  |  |  |  |  |  |  |  |  |  |  |  |
| Do you go out less frequently compared with last year? | Yes | 211 | 1.08 | (0.96, 1.21) | 0.21 |  | 84 | (82, 86) | 0.42 |  | 0.812 | (0.778, 0.846) | 0.30 |  | 21.3 | (20.5,22.1) | 0.13 |
|  | No | 99 | 1.00 | (Reference) |  |  | 86 | (83, 88) |  |  | 0.844 | (0.794, 0.893) |  |  | 22.3 | (21.2, 23.5) |  |
|  |  |  |  |  |  |  |  |  |  |  |  |  |  |  |  |  |  |
| Do you sometimes visit your friend? | Yes | 108 | 1.00 | (Reference) | 0.11 |  | 84 | (82, 86) | 0.42 |  | 0.881 | (0.834, 0.928) | < 0.01 |  | 22.8 | (21.7, 24.0) | < 0.01 |
|  | No | 202 | 1.10 | (0.98, 1.23) |  |  | 86 | (83, 88) |  |  | 0.790 | (0.755, 0.824) |  |  | 20.9 | (20.1, 21.8) |  |
|  |  |  |  |  |  |  |  |  |  |  |  |  |  |  |  |  |  |
| Do you feel you are helpful to friends or family? | Yes | 229 | 1.00 | (Reference) | < 0.01 |  | 87 | (85, 89) | < 0.01 |  | 0.864 | (0.832, 0.895) | < 0.01 |  | 22.5 | (21.8, 23.3) | < 0.01 |
|  | No | 81 | 1.25 | (1.10, 1.42) |  |  | 78 | (75, 81) |  |  | 0.706 | (0.652, 0.759) |  |  | 19.0 | (17.7, 20.3) |  |
|  |  |  |  |  |  |  |  |  |  |  |  |  |  |  |  |  |  |
| Do you Live alone? | Yes | 64 | 1.00 | (0.87, 1.15) | 0.99 |  | 84 | (81, 88) | 0.81 |  | 0.873 | (0.812, 0.935) | 0.07 |  | 22.2 | (20.7, 23.7) | 0.38 |
|  | No | 246 | 1.00 | (Reference) |  |  | 85 | (83, 86) |  |  | 0.808 | (0.776, 0.840) |  |  | 21.4 | (20.7, 22.2) |  |
|  |  |  |  |  |  |  |  |  |  |  |  |  |  |  |  |  |  |
| Do you talk with someone everyday? | Yes | 274 | 1.00 | (Reference) | 0.36 |  | 85 | (83, 86) | 0.76 |  | 0.818 | (0.788, 0.848) | 0.49 |  | 21.6 | (20.9, 22.3) | 0.88 |
|  | No | 36 | 0.92 | (0.78, 1.09) |  |  | 85 | (81, 90) |  |  | 0.849 | (0.767, 0.931) |  |  | 21.5 | (19.5, 23.4) |  |

Models were constructed using logistic regression analysis and analysis of covariance and were adjusted for age and sex. Abbreviations: OR, odds ratio; CI, confidence interval; LSM, least square mean.


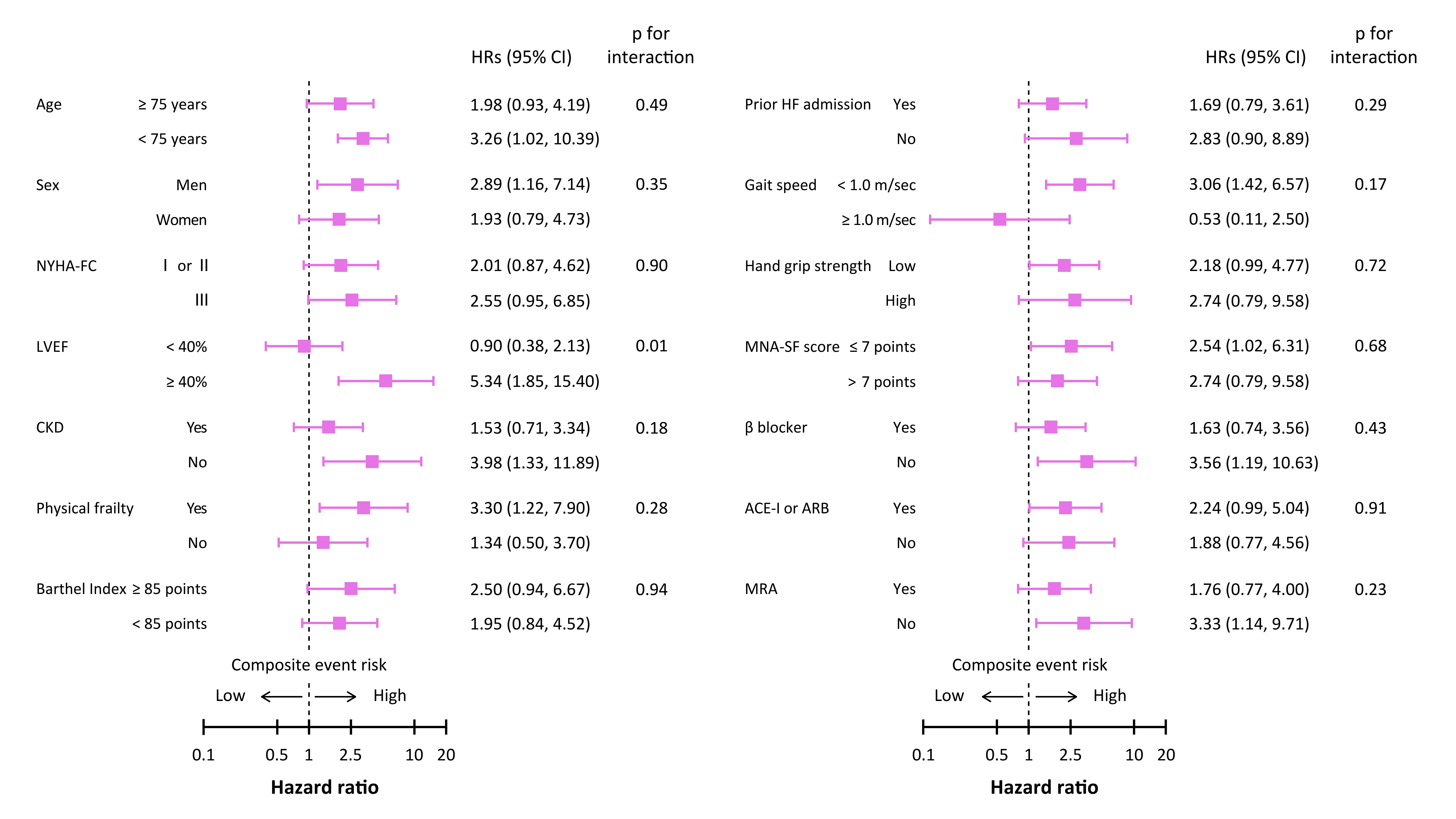


**Supplementary Figure 1.** Forest plots of subgroup analyses of the associations between social frailty and composite events.

All subgroups were adjusted for age, sex, log NT-proBNP, and Charlson comorbidity index. Abbreviations: HR, hazard ratio; CI, confidence interval ; NYHA-FC, New York Heart Association functional class; LVEF, left ventricular ejection fraction; CKD, chronic kidney disease; HF, heart failure; MNA-SF, mini nutritional assessment short form; NT-proBNP, N-terminal pro B-type natriuretic peptide; ACE-I, angiotensin-converting enzyme inhibitor; ARB, angiotensin receptor blocker; MRA, mineralocorticoid receptor antagonist.
